# High Submandibular Anteroparotid Approach for Open Reduction and Internal Fixation of Condylar Fracture

**DOI:** 10.1155/2021/5542570

**Published:** 2021-07-09

**Authors:** Kamichika Hayashi, Takeshi Onda, Hirona Honda, Mitsuru Takata, Hiroyuki Matsuda, Hidetoshi Tamura, Masayuki Takano

**Affiliations:** ^1^Department of Oral and Maxillofacial Surgery, Tokyo Dental College, Tokyo, Japan; ^2^Oral and Maxillofacial Surgery, Kameda General Hospital, Chiba, Japan

## Abstract

**Aim:**

There are several techniques for the treatment of mandibular condylar fractures. This is the first report of the high submandibular anteroparotid approach for open reduction and internal fixation of condylar fracture.

**Materials and Methods:**

A 41-year-old woman fell indoors and injured her face. She was referred to our department for detailed examination and treatment of a suspected mandibular fracture. X-ray and computed tomography showed a right mandibular condylar base fracture and lateral dislocation of the fracture fragment. Open reduction and internal fixation procedures were performed for a right mandibular condylar fracture under general anesthesia. The mandibular ramus was reached by approaching from the inferior margin of the mandible, delaminating the masseter fascia posteriorly, and bypassing the anterior margin of the parotid gland. Once the fractured bone was reached, reduction and fixation were performed.

**Results:**

We have achieved good results by the high submandibular anteroparotid approach, which is minimally invasive and simple, to reduce and fix condylar fractures. With this approach, no facial artery or retromandibular vein was encountered, and the mental stress for the surgeon was minimal. Postoperative wound infection, parotid gland complications such as parotitis and salivary fistula, facial nerve dysfunction such as facial paralysis, and esthetic disorders such as scarring were not observed.

**Conclusions:**

Although it is necessary to examine more cases in the future, the high submandibular anteroparotid approach may be useful as a new approach for open reduction and internal fixation of condylar fractures.

## 1. Introduction

The treatment policy for mandibular condylar fractures is based on local factors, such as the fracture site and the presence or absence of concurrent fractures, and patient characteristics such as age, general condition, and social background [1, 2]. There is no consensus on the treatment for mandibular condylar fractures [3–5]. In adult mandibular condylar neck and base fractures, surgical therapy is reported to have a better functional prognosis than nonsurgical therapy [6–9].

The approaches to surgery can be categorized mainly as intraoral and extraoral. The intraoral approach is performed using an intraoral mucosal incision. Extraoral approaches include the Al-Kayat approach performed using a preauricular incision and the retromandibular approach and submandibular approaches (the Risdon approach) performed using skin incisions. The choice of surgery should be based on the fracture site (head, neck, and base of the mandibular condyle) and mode (crack, displacement, metastasis, or dislocation) [10]. Both approaches consider the course of the facial nerve. Recently, the clinical usefulness of the retromandibular approach [11–13], the downward and curvilinear extension of the preauricular skin crease incision [14–16], and the high perimandibular transmasseteric approach (HPTM) [17–20] have been recognized. These approaches facilitate the expansion of the operative field for mandibular condylar neck and base fractures and minimize the risk of postoperative facial nerve dysfunction.

We applied a high submandibular anteroparotid approach involving a skin incision in the inferior margin of the mandible to the delamination of the masseter fascia in the posterior direction, by bypassing the anterior margin of the parotid gland to reach the mandible and obtained good results. A summary of a case is presented along with a literature review.

## 2. Case Presentation

**Figure 1:**
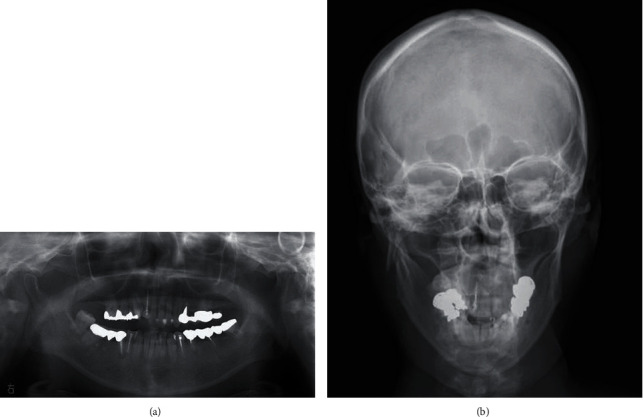
(a) Panoramic X-ray at the first visit. A right mandibular condylar base fracture is observed. (b) A simple head X-ray at the first visit (P-A). A fracture of the right mandibular condyle and external displacement of the bone fragment are observed.

**Figure 2:**
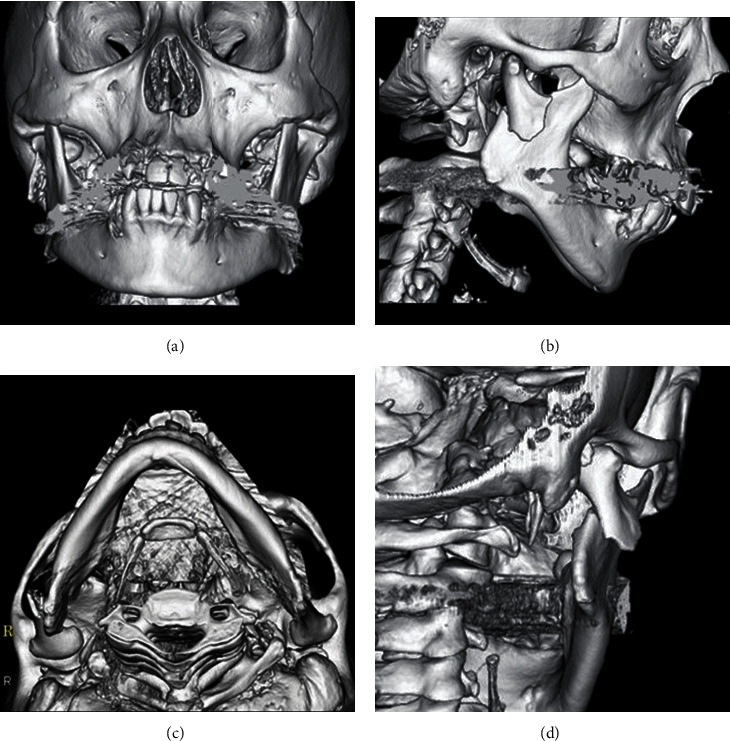
3D-constructed CT image at the first visit. (a) Front. (b) Right side. (c) Axial. (d) 45 degrees behind to the right. CT imaging shows a right mandibular condylar base fracture and lateral dislocation of the fracture fragment. No fractures are observed except for those of the mandible. No fractures of the alveolar bones of the upper and lower jaws are also observed. No tooth fracture is observed.

The patient was a 41-year-old woman with a height of 166 cm and a weight of 58 kg. In January 2019, she fell indoors and injured her face and visited a hospital for medical treatment. Her chief complaint was trismus. A close examination of the head at the head and neck surgery department showed no abnormal findings. She was referred to our department for detailed examination and medical treatment for a suspected mandibular fracture. Her first visit to our department was on the following day. Her family history was unremarkable. Facial findings on the first visit included mild swelling and reddening in the right preauricular region. The intraoral finding was displacement of the median line of the mandible to the right. The right molar was also in early contact, and the left molar had an open bite. Trismus was observed, and her mouth opening capacity was 10 mm between the upper and lower central incisors. A panoramic radiograph showed a right mandibular condylar base fracture (subcondylar fracture, Lindahl's classification [21]) (Figure 1(a)). An X-ray of the head (P-A) showed a right mandibular condylar dislocation fracture (displacement fracture, Maclennan's classification [22]) (Figure 1(b)). Computed tomography (CT) imaging showed a right mandibular condylar base fracture and lateral dislocation of the fracture fragment (Figure 2). No fractures were observed elsewhere. No fractures of the teeth and of the alveolar bones of the upper and lower jaws were seen. Amoxicillin 750 mg/day was prescribed for 3 days to prevent infection, and acetaminophen 500 mg was prescribed for pain on an as-needed basis. Because she hoped for early functional recovery, open reduction and internal fixation, rather than a nonsurgical approach, were chosen. The patient was consulted on the treatment policy.

**Figure 3:**
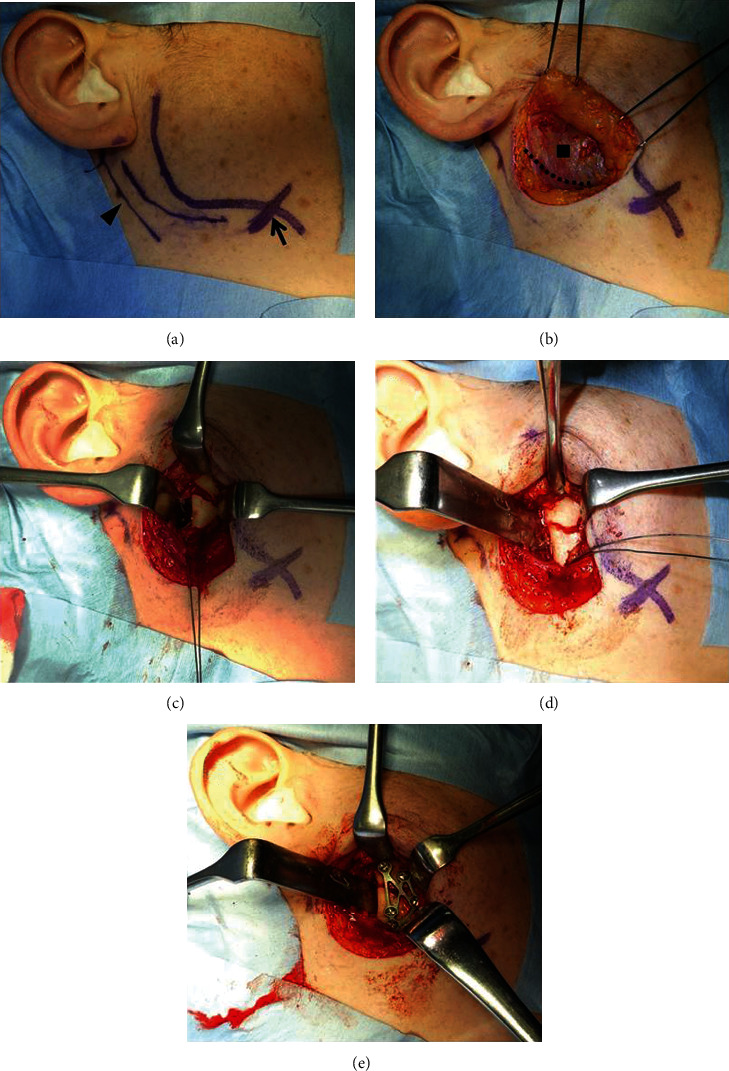
(a) Black arrow: facial artery; black triangle: sternocleidomastoid muscle. The skin incision line, the outline of the mandible, the sternocleidomastoid muscle, and the facial artery are traced using gentian violet. The planned skin incision is 0.5 mm caudally from the mandibular margin and 5 to 7 mm posteriorly from the posterior margin of the ramus to include the mandibular angle. An incision line with a total length of approximately 5 cm is made. (b) Black dotted line: platysma incision line; black square: masseter muscle. The platysma fascia is incised, approximately 2 cm subcutaneously, and the flap is raised. Once the platysma is located, it is incised to the depth of the masseter fascia such that the incision is 1 cm cranial from the mandibular margin in the anterior direction and 2 cm cranial from the mandibular margin in the posterior direction. In women, the platysma is thin, and the masseter fascia lies immediately inside the platysma. (c) A reverse warping muscle retractor is applied to the posterior margin of the ramus to gently extrude the parotid gland backward, and the masseter muscle is pulled slightly forward with the muscle retractor to expand the operative field. The periosteum is delaminated from the posterior margin of the ramus using a raspatorium, and the fracture site is located. A 0.5-mm-diameter metal wire for pulling the mandibular ramus is passed through caudally to approximately 2 cm of the fracture line. (d) By pulling the metal wire downward, the ramus is pulled sufficiently downward, and reduction is achieved. (e) After reduction, the fragment is fixed using a MatrixMANDIBLE Subcondylar Strut Plate.

Nine days after the first visit, open reduction and internal fixation procedures were performed on the right mandibular condylar fracture under general anesthesia. During surgery, 1 g of cefmetazole sodium was administered to prevent surgical site infection. The skin incision was designed to be 5 mm caudal to the inferior margin of the mandible and 5 to 7 mm posterior to the posterior margin of the mandible to include the mandibular angle. The incision line extended more posteriorly than Wilk's skin incision [17, 18]. Thus, an incision line with a total length of approximately 5 cm was made (Figure 3(a)). Subsequently, a subcutaneous cut of approximately 2 cm was made in the skin along the platysma fascia. Delamination was performed between the subcutaneous and platysma fascia. The platysma was located and incised to the depth of the masseter fascia; the incision was 1 cm cranial from the mandibular margin in the anterior direction and 2 cm cranial from the mandibular margin in the posterior direction. Wilk and Biotchane [17, 18] made an incision up to the masseter muscle; however, in the present case, an incision was made only in the platysma muscle, and the masseter fascia was delaminated approximately 2 cm further cranially along the masseter fascia. The platysma was thin, with the masseter fascia immediately inside the platysma (Figure 3(b)). A buccal muscle branch that was running along the masseter fascia was found and retracted cranially to avoid injuring it. Next, the masseter fascia was delaminated towards the posterior margin of the ramus. While proceeding with the delamination towards the posterior margin of the ramus, the parotid gland was encountered, and delamination was performed between the parotid gland and the masseter fascia using a Metzenbaum scissor. A muscle retractor was applied to the anterior margin of the parotid gland to slight traction of the parotid tissue backward. The posterior margin of the parotid gland was located by inserting a reverse warping muscle retractor at the posterior margin of the ramus followed by a gentle displacement of the parotid gland posteriorly. A periosteal incision was made in the posterior margin of the ramus by a round-edged knife. Next, the periosteum on the medial and lateral surfaces of the ramus was delaminated by a periosteal elevator, and the fracture site was reached. A muscle retractor was inserted on the anterior side, and the masseter muscle was slightly extruded anteriorly to permit the localization and observation of the fractured site. A protector was inserted inside the mandibular ramus; a hole of approximately 2 cm was drilled from the lateral side on the caudal side of the fracture line by a steel bar. A 0.5-mm-diameter metal wire was passed through the hole (Figure 3(c)). An assistant attempted to reduce the fractured fragment by pulling the metal wire and the ramus downward (Figure 3(d)). After reducing the fractured fragment, plate fixation was performed. A MatrixMANDIBLE Subcondylar Strut Plate (Depuy Synthes, Switzerland), developed for mandibular condylar fractures was used for the fixation (Figure 3(e)) [23]. The surgeon manually confirmed the patient's jaw movements. The surgeon manually opened and closed the patient's jaw. In addition, the surgeon manually moved the patient's jaw from side to side. And after confirming the absence of occlusion, a periosteal suture was placed with an absorbable thread. The platysma was also sutured, followed by the dermis. The skin was sutured with a nonabsorbable thread. Finally, Dual-Top anchor screws (Jeil Medical Corporation, Korea) were placed in the maxilla and mandible for postoperative intermaxillary traction, and that completed the procedure. There were no abnormalities during the surgical procedure.

**Figure 4:**
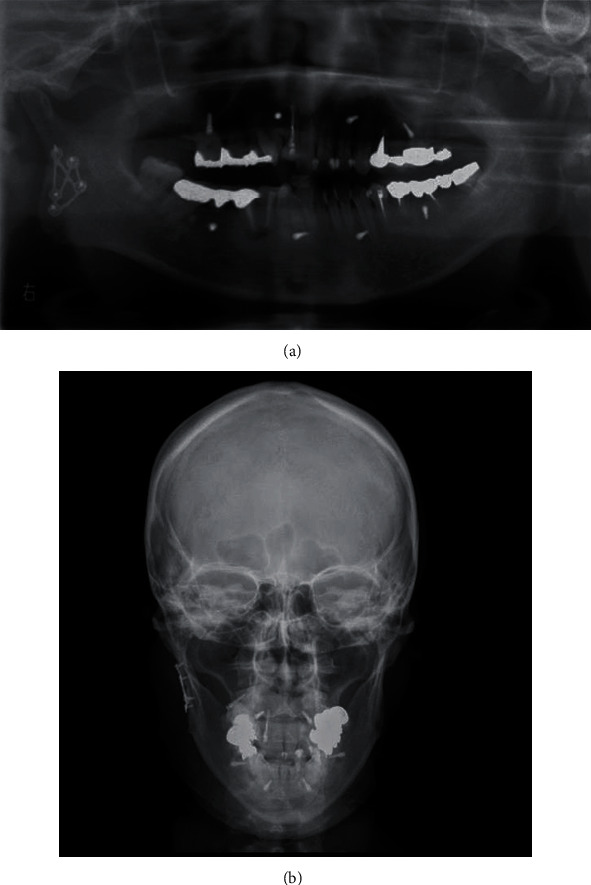
(a) Panoramic X-ray on the day after the surgery. The right mandibular condylar fracture is reduced and fixed. (b) Head X-ray (PA) on the day after surgery. The bone fragment that was displaced externally is reduced and fixed.

Cefmetazole sodium 2 mg/day was administered for 48 hours after surgery to prevent postoperative infection, and acetaminophen 1000 mg was prescribed for pain on an as-needed basis. The reduction and fixation were confirmed on panoramic and head X-rays (P-A) a day after the surgery (Figures 4(a) and 4(b)). No postoperative dysfunction such as facial paralysis was observed. From the day after surgery, elastic was used for intermaxillary fixation to control mouth opening. Two weeks after the surgery, the intermaxillary fixation was released. She started self-opening exercises involving daily horizontal movements of the lower jaw forward and to the left and right and vertical movements involving opening and closing of the mouth. Approximately one month after the surgery, her mouth opening capacity recovered to 30 mm between the upper and lower central incisors. By 2 months after the surgery, her mouth opening capacity had recovered to 40 mm. Three months after the surgery, she recovered and had no interference with her daily life. No facial deformity, wound infection, or scarring was observed.

## 3. Discussion

Mandibular fractures are the most common facial fractures. Mandibular condylar fractures are the most prevalent of mandibular fractures, accounting for 25-50% of all jaw fractures [24, 25]. Treatment policies for condylar fractures vary from institution to institution, and no gold standard for treatment has been determined [3, 4]. Recently, open reduction and internal fixation surgery have been recommended for adult condylar fractures from the perspective of functional recovery [7–9, 26–29]. When applying open reduction and internal fixation surgery for a condylar fracture, the approaches to the condyle are generally classified as intraoral and extraoral. The intraoral approach is associated with superior aesthetics and a low risk of nerve damage; however, it is only applicable to a limited number of cases. When it is used, it is difficult to expand the operative field, and the narrow field of view makes it difficult to reach the fracture site and operate [30]. Therefore, the intraoral approach can be applied only to condylar base fractures that are fissure fractures or just slightly lateral displacement/dislocation fractures. It should not be applied otherwise. Moreover, due to its difficulty, its duration is longer than that of the extraoral approach [31]. Recently, an intraoral approach using an endoscope has been reported; however, its use has not been widespread because an endoscope is required [32]. The extraoral approaches include the following, ordered from the cranial to the caudal and depending on the position of the skin incision: the Al-Kayat approach [33], preauricular skin crease incision extended downward in a curvilinear manner [14], the retromandibular approach [11, 12], HPTM [18], and the Risdon approach [34]. These approaches also have advantages and disadvantages. Which approach to the fractured site to select has not been determined [10, 31, 35–38].

In open reduction and internal fixation surgery for mandibular condylar fractures, the greatest concern is a facial nerve disorder. Surgeons need to consider the course of the facial nerve when approaching the articular process [39]. The facial nerves form the parotid plexus on the masseter muscle. An anastomosis of the buccal and zygomatic branches is seen in 70 to 100% of cases, whereas temporal branches and ramuses are isolated peripheral branches with few nerve anastomoses [40]. Temporal branches and ramuses may be involved in postoperative dysfunction following excessive traction and compression associated with the surgery. The traffic between the marginal ramus and branches of other facial nerves is as low as 0-16% [20, 40]. There is a high risk of persistent postoperative nerve palsy occurring in the temporal branch with the Al-Kayat approach [33] and in the marginal ramus with the Risdon approach [32]. Recently, the retromandibular approach (RMRP) [11–13, 41], preauricular skin crease incision extended downward in a curvilinear manner [14–16], retromandibular transparotid approach (RMTP) [15, 42–45], retroparotid transmasseteric approach (RPTM), and transmasseteric anteroparotid approach (TMAP) [46, 47] and the HPTM [18] have been commonly used for mandibular condylar neck and base fractures.

**Figure 5:**
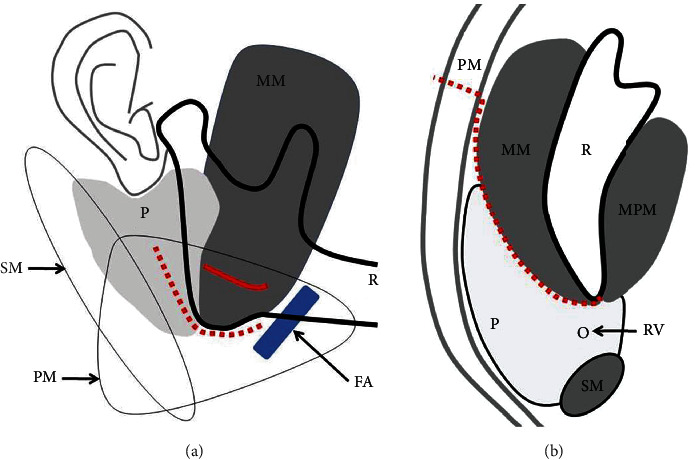
(a) FA: facial artery; MM: masseter muscle; P: parotid glands; PM: platysma; R: mandibular bone; SM: sternocleidomastoid muscle; red dotted line: skin incision line; red line: platysma incision line. (b) MM: masseter muscle; P: parotid glands; MPM: medial pterygoid muscle; PM: platysma; R: mandibular bone; RV: retromandibular-vein; SM: sternocleidomastoid muscle; Red dotted line: the approach route.

In the high submandibular anteroparotid approach, a skin incision is made immediately below the inferior margin of the mandible into the platysma on the cranial side of the marginal ramus of the facial nerve; this minimizes the risk of directly injuring the marginal ramus. As the distance to the articular process is short, and the amount of tissue that is pulled cranially when expanding the operative field is small, the risk of damage to the mandibular bifurcation following intraoperative traction and compression is low. In the high submandibular anteroparotid approach, it is easy to identify the buccal branch that runs along the masseter fascia, and it should be protected by retracting it cranially or caudally when it is exposed. Even if the buccal branch is injured, the likelihood of anastomoses with other nerve branches is high, and postoperative facial nerve disorders are therefore unlikely. In addition, the skin incision immediately below the mandibular margin in the high submandibular anteroparotid approach is inconspicuous and hidden within the mandibular margin. Since the skin traction during surgery is minimal, postoperative scarring is also minimal. This is an approach that allows adequate expansion of the operative field, and optimal reduction and fixation can be performed without extensive pulling of the surrounding tissue. Wilk's approach was improved by dissecting only the platysma and advancing the masseter fascia posteriorly, thereby bypassing the anterior margin of the parotid gland to reach the posterior margin of the ramus. With this improved approach, no facial artery or retromandibular vein was encountered, and the mental stress for the surgeon was minimal [46, 47]. (Figures 5(a) and 5(b)).

In patients with developed parotid glands and a large amount of fat, the soft tissues expand, which makes it difficult to expand the operative field. In addition, it is difficult to distinguish the boundary between the anterior margin of the parotid gland and adipose tissue in a patient with parotid atrophy from fatty degeneration due to aging. The high submandibular anteroparotid approach has these drawbacks, and it may take time to learn.

In the present case, the mandibular condylar fracture was fixed with an adequate operative field permitted by the high submandibular anteroparotid approach. Postoperative surgical site infection, parotid gland complications such as parotitis and salivary fistula, facial nerve dysfunction such as facial paralysis, and esthetic disorders such as scarring were not observed. Although it is necessary to examine more cases in the future, the high submandibular anteroparotid approach may be useful as a new approach for open reduction and internal fixation of condylar fractures.

## 4. Conclusion

Open reduction and internal fixation procedures were performed for a right mandibular condylar fracture. The ramus was reached by approaching from the inferior margin of the mandible, delaminating the masseter fascia posteriorly, and bypassing the anterior margin of the parotid gland. Once the fractured bone was reached, reduction and fixation were performed. Satisfactory results were obtained. After the surgery, no functional impairment was observed.
